# The association between KLF4 as a tumor suppressor and the prognosis of hepatocellular carcinoma after curative resection

**DOI:** 10.18632/aging.103592

**Published:** 2020-08-05

**Authors:** Min Xue, Chenhao Zhou, Yan Zheng, Ziping Zhang, Shun Wang, Yan Fu, Manar Atyah, Xiaolong Xue, Le Zhu, Qiongzhu Dong, Huliang Jia, Ning Ren, Ruolei Hu

**Affiliations:** 1 Department of Biochemistry and Molecular Biology, Laboratory of Molecular Biology, Anhui Medical University, Hefei, China; 2 Department of Liver Surgery, Liver Cancer Institute, Zhongshan Hospital, Fudan University, Shanghai, China; 3 Department of General Surgery, Huashan Hospital and Cancer Metastasis Institute, Fudan University, Shanghai, China; 4 Institute of Fudan Minhang Academic Health System, Minhang Hospital, Fudan University, Shanghai, China

**Keywords:** KLF4, hepatocellular carcinoma, prognosis, overall survival, recurrence-free survival

## Abstract

Krüppel-like factor 4 (KLF4), a zinc-finger transcription factor in klfs family, is known for its crucial role in regulating cell growth, proliferation, and differentiation. This research aimed to explore the prognostic significance of KLF4 in hepatocellular carcinoma’s (HCC) patients after curative resection and the role of KLF4 in HCC progression. There were 185 HCC patients who had hepatectomy from July 2010 to July 2011 included in this study. KLF4 expression was detected by microarray immunohistochemical technique, western blot, and qRT-PCR. Then, the correlation between the prognosis of patients and KLF4 expression was evaluated based on patients’ follow-up data. The research found KLF4 expression was significantly downregulated in HCC tissues compared to para-tumorous tissues. More importantly, the overall survival rate (OS) and recurrence-free survival rate (RFS) of HCC patients with low KLF4 expression were both significantly decreased compared to those with a high level of KLF4. Further function and mechanism analysis showed that KLF4 could inhibit the proliferation, migration, invasion and epithelial-mesenchymal transition of HCC cells. The study revealed that KLF4 was not only a tumor suppressor in HCC but also can be regarded as a valuable prognostic factor and potential biological target for diagnosis and treatment in HCC patients.

## INTRODUCTION

Hepatocellular carcinoma (HCC) is one of the most common types of cancer and ranks second in the leading causes of cancer-related death worldwide [1, 2]. Despite the great achievement in HCC treatment, the mortality in HCC patients remains high, which may be attributed to frequent tumor recurrence and distant metastasis. In addition to the critical role of early diagnosis and intervention in HCC treatment, postoperative monitoring also plays a significant role in improving HCC patients’ prognosis [3, 4]. However, there is a lack of prognosis markers for HCC patients receiving a hepatectomy, necessitating the investigation of clinically useful biomarkers for those patients.

Furthermore, due to the low early detection rate and complicated risk factors, the diagnosis of most HCC patients often occurs at an advanced stage with poor prognosis. Considering current medical capabilities, curative liver resection or radiofrequency ablation (RFA) is the first choice for early-stage HCC, but it is restricted by many conditions such as age, tumor location, bilirubin level < 1.0mg/dL, and hepatic venous pressure < 10 mm Hg. The patients with HCC at early-stage received resection, liver transplantation or RFA with a 50–70% 5-year survival rate [5, 6]. Hence, it is important to explore more alternative clinical biomarkers, which can enhance the diagnosis and treatment in all stages of HCC. Not only radical surgery but also liver transplantation are the most essential treatments provided to patients diagnosed at early stages. Despite the emergence of some drug trials such as Sorafenib and Brivanib, no enhancement of the overall survival rate has been achieved yet [7–10]. With the development of tumor progression, HCC treatment faces a lot of challenges, especially in advanced stages of the disease. Therefore, it is urgent to explore latent molecular mechanisms of HCC progression, which would improve surgical treatment and prognosis of HCC patients.

The transcription factor Krüppel-like factor 4 (KLF4) with zinc-finger structure has specific binding sites and is a member of SP/KLF factors’ family. It can regulate cell growth, proliferation, and differentiation in the process of development [11, 12]. In different kinds of tissues, KLF4 plays different roles, either acting as an oncogene or as a tumor suppressor [13, 14]. In cervical carcinoma, KLF4 as a tumor suppressor inhibited cell growth and tumor formation [15]. In lung cancer, KLF4 negatively regulated placenta-specific 8 (PLAC8) expression by binding to the promoter of PLAC8, which suppressed cell proliferation and apoptosis [16]. In addition, KLF4 negatively regulated podocalyxin-like 1 (PODXL) expression to inhibit human gastric cancer’s tumorigenesis, invasion, and metastasis [17]. Judging from clinical and experimental data, KLF4 has an anti-cancer effect on suppressing tumor differentiation, proliferation, invasion, epithelial-mesenchymal transition (EMT), and metastasis [18–20]. In addition, recent studies have shown that KLF4 is a key regulator of monoglyceride lipase (MGLL) and plays an essential role in inhibiting HCC cell migration [21]. KLF4 can elevate miR-153, miR-506 and miR-200b levels to downregulate EMT-associated proteins [22]. Epithelial-mesenchymal transition (EMT) is a process of the transformation of epithelial phenotype to a mesenchymal phenotype. Multiple lines of evidence have demonstrated that EMT was strongly associated with cancer cell proliferation, invasion and metastasis [23, 24]. Recent studies have shown that KLF4 is a key negative regulator of EMT, and the expression of KLF4 is often decreased during the process of EMT [25]. In addition, E-cadherin was found to be the target gene of KLF4 in invasion and metastasis of breast cancer [26]. However, the prognostic value of KLF4 in HCC patients and the influencing mechanism of KLF4 in the progression of HCC have been rarely explored.

In our research, we explored the prognostic values of KLF4 by analyzing the correlation between KLF4 and clinicopathologic features in HCC patients. In addition, we investigated the functions of cells by overexpressing and knocking-down KLF4 and the regulating effects of KLF4 on EMT in HCC cells. We determined KLF4 as an independent prognostic biomarker for HCC patients, and the inhibiting effect of KLF4 on EMT is expected to provide new therapeutic targets.

## RESULTS

### Different patterns were used for detecting KLF4 expression in HCC

**Figure 1:**
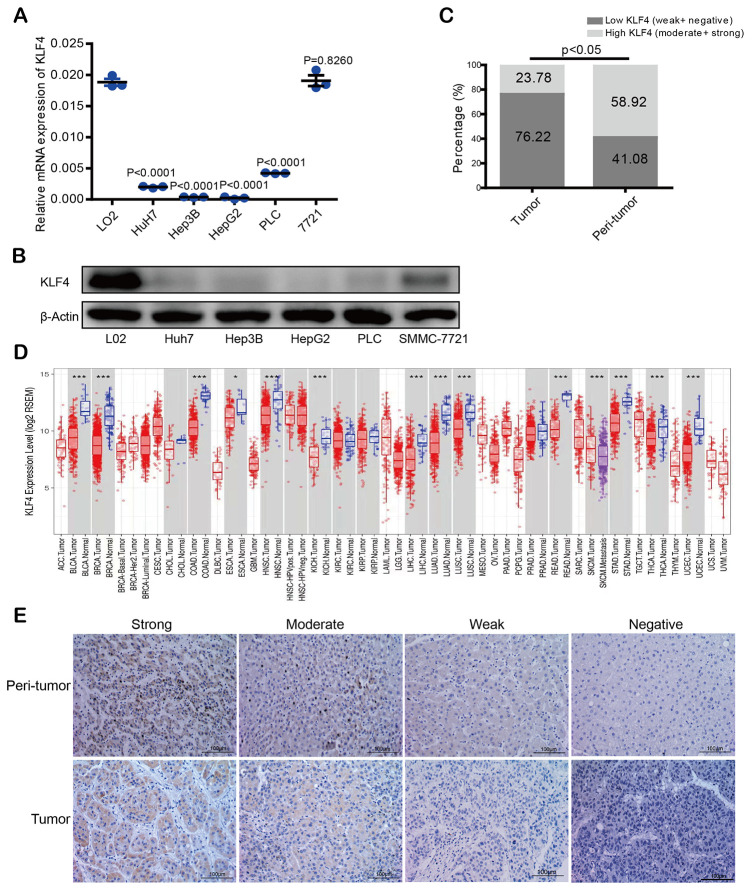
**KLF4 expression in hepatocellular carcinoma (HCC) tissues and cell lines.** (**A**) KLF4 expression was detected in the mRNA level among five HCC cell lines and one normal liver cell (L02). (**B**) KLF4 expression was detected in the protein level in six cell lines. The internal control was β-actin. (**C**) Immunohistochemical results were analyzed by chi-square test to compare the distribution of KLF4 in HCC tumors and adjacent tissues. (**D**) KLF4 expression was analyzed in tumor and para-tumorous tissues in TCGA tumors. (**E**) KLF4 expression was exhibited through characteristic photos of immunostaining in HCC tumor and para-tumorous tissues. Image scale= 100μm. *P* < 0.05 was considered statistically significant, * *P* < 0.05, ** *P* < 0.01, *** *P* < 0.001.

KLF4 expression in most HCC cells (PLC/PRF/5, Hep3B, Huh7, HepG2, but except SMMC- 7721) was lower than in normal liver cells (L02) not only at mRNA level but also at the protein expression level (Figure 1A and 1B). Same KLF4 expression patterns were found in the TCGA database (https://tcga-data.nci.nih.gov/tcga/), with lower expression observed in many kinds of tumors including lung adenocarcinoma (LUAD), bladder urothelial carcinoma (BLCA), and cholangiocarcinoma (CHOL) (opposing to matched para-tumorous tissues) (Figure 1D).

Immunohistochemical staining intensity in a tissue microarray (TMA) was divided into four levels, which is our scoring criterion for KLF4 expression in tissues (Figure 1E). According to the score statistics of staining degree and the staining area, high KLF4 expression rate in para-tumorous tissues was 58.92% (109 of 185: 16 of strong and 93 of moderate) while high KLF4 expression rate in HCC tissues was 23.78% (44 of 185: 4 of strong and 40 of moderate), which showed KLF4 expression in tumor tissues is lower than the paired para-tumorous tissues (*P <0.05) (Figure 1C). The results demonstrated that KLF4 may be a potential prognostic marker for those patients with HCC.

### KLF4 expression is related to some clinicopathological features

Depending on the scores of immunohistochemical staining, 185 HCC patients were distributed into two groups: one group had high KLF4 expression and the other group had low KLF4 expression. High KLF4 expression group showed a statistical score of strong and moderate staining while the low KLF4 expression group showed a statistical score of weak and negative staining. Table 1 shows that low KLF4 expression was associated with many clinical phenotypes, such as vascular invasion (***P <0.001), high alpha-fetoprotein level (*P = 0.012), and advanced Barcelona Clinic Liver Cancer (BCLC) stage (**P = 0.002). There was no remarkable correlation between other clinical characteristics and KLF4 in our research.

**Table 1:** KLF4 expression in 185 HCC patients based on clinicopathologic characteristics.

**Characteristics**	**Patients**	**KLF4 expression**		***P***
	**n (%)**	**Low (n = 141)**	**High (n = 44)**	
Age, years				
≤ 50	94 (50.8)	69	25	
> 50	91 (49.2)	72	19	0.392
Gender				
Female	31 (16.8)	21	10	
Male	154 (83.2)	120	34	0.250
HBsAg				
Negative	26 (14.1)	20	6	
Positive	159 (85.9)	121	38	1.000
AFP, ng/ml				
≤ 20	70 (37.8)	46	24	
> 20	115 (62.2)	95	20	0.012*
Liver cirrhosis				
NO	29 (15.7)	21	8	
Yes	156 (84.3)	120	36	0.637
Tumor number				
Single	167 (90.3)	124	43	
Multiple	18 (9.7)	17	1	0.078
Tumor size, cm				
≤ 5	130 (70.3)	96	34	
> 5	55 (29.7)	45	10	0.264
Vascular invasion				
Absent	121 (65.4)	81	40	
Present	64 (34.6)	60	4	< 0.001***
Tumor differentiation				
Ι-II	136 (73.5)	99	37	
III-IV	49 (26.5)	42	7	0.080
BCLC stage				
0+A	39 (21.1)	22	17	
B+C	146 (78.9)	119	27	0.002**
Tumor capsule				
Complete	101 (54.6)	73	28	
None	84 (45.4)	68	16	0.225
ALT, U/L				
≤40	170 (91.9)	129	41	
>40	15 (8.1)	12	3	1.000

Abbreviations: HCC, hepatocellular carcinoma; HBsAg, hepatitis B surface antigen; AFP, α- fetoprotein; BCLC, Barcelona Clinic Liver Cancer; ALT, alanine aminotransferase. *P* < 0.05 was considered statistically significant, Pearson χ2 tests. * *P* < 0.05, ** *P* < 0.01, *** *P* < 0.001.

### The level of KLF4 expression was related with OS and RFS of HCC patients

**Figure 2:**
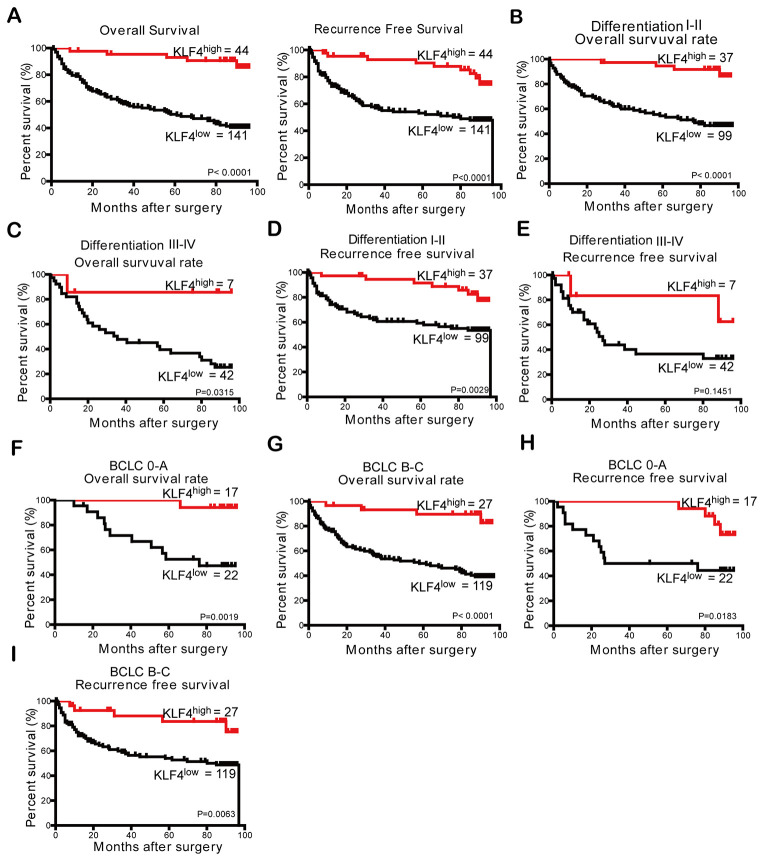
**The prognostic analysis of KLF4 in HCC patients (n = 185) and subgroup analysis based on differentiation degree and BCLC stages.** (**A**) According to KLF4 expression level in patients’ tissues with HCC, the curves described OS and RFS of patients, respectively. The OS in (**B**) differentiation I-II patients and (**C**) differentiation III-IV patients was analyzed by Kaplan- Meier method. The RFS in (**D**) differentiation I-II patients and (**E**) differentiation III-IV patients were analyzed by Kaplan- Meier method. Kaplan- Meier method was employed to analyze the OS of patients in (**F**) BCLC 0-A group and (**G**) BCLC B-C group. The RFS of patients in two subgroups (**H** and **I**) of the BCLC stage was analyzed through Kaplan- Meier analysis. P < 0.05 was considered statistically significant.

The results showed a relation between the outcome of HCC and KLF4 expression when evaluating OS and RFS of patients. Figure 2A showed HCC patients with high KLF4 expression in tumor tissues displayed a favorable prognosis in OS (OS, ***P <0.001) and RFS (RFS, ***P <0.001) analyses compared to those patients with low KLF4 expression.

From the analysis of the impact of different differentiation degree or BCLC stages on OS and RFS, we can see patients with higher KLF4 expression in “differentiation I-II” subgroups had a better OS (***P <0.001, Figure 2B) and RFS (**P =0.0029, Figure 2D). However, differences in KLF4 expression of the “differentiation III-IV” subgroup failed to predict similar outcomes (Figure 2C and 2E). Then to further analyze the relationship between KLF4 and prognosis in different BCLC subgroups, results show the patients in all stages of BCLC with low KLF4 expression group had shorter OS (**P = 0.0019, Figure 2F and ***P <0.0001, Figure 2G) and RFS (*P =0 .0183, Figure 2H and Figure 2I, **P =0.0063).

### Low expression of KLF4 may mean poor prognosis for HCC patients

Table 2 showed the prognostic role of KLF4 in HCC patients. In univariate analysis, tumor size and the degree of tumor differentiation were significantly related to HCC patients’ OS and RFS. The OS was also significantly related to tumor number and microvascular invasion while RFS was also significantly associated with serum HBsAg level. However, other features including age, gender, ALT, liver cirrhosis, tumor encapsulation, and serum AFP level showed no significant prognostic associations with OS or RFS. A multivariate Cox regression analysis was then conducted with all the prognostic factors of *P < 0.05 in univariate analysis. Based on the results, we concluded high KLF4 was identified as a positive prognostic factor for OS and RFS of HCC patients.

**Table 2:** The prediction for OS and RFS of 185 HCC patients with univariate and multivariate analyses.

**Variables**	**OS**			**RFS**		
	**Univariate**	**Multivariate**		**Univariate**	**Multivariate**	
	***P***	**HR (95% CI)**	***P***	***P***	**HR (95% CI)**	***P***
Age (> 50 vs. ≤ 50)	0.619		NA	0.444		NA
Gender (male vs. female)	0.326		NA	0.756		NA
ALT, U/L (> 40 vs. ≤ 40)	0.462		NA	0.946		NA
AFP, ng/mL (> 20 vs. ≤ 20)	0.061		NA	0.488		NA
HBsAg (positive vs. negative)	0.276		NA	0.032	2.836 (1.138-7.067)	0.025
Liver cirrhosis (yes vs. no)	0.369		NA	0.169		NA
Tumor size, cm (> 5 vs. ≤ 5)	0.003	1.731 (1.101-2.722)	0.018	0.021	1.671 (1.017-2.746)	0.043
Tumor number (multiple vs. single)	0.004		NS	0.215		NA
Tumor differentiation (III-IV vs. I-II)	0.007		NS	0.010		NS
Tumor encapsulation (complete vs. none)	0.093		NA	0.090		NA
Vascular invasion (present vs. absent)	0.001		NS	0.402		NA
KLF4 (Low vs. High)	< 0.001	5.959 (2.366-15.004)	< 0.001	0.001	3.477 (1.720-7.027)	0.001

Abbreviations: OS, overall survival; RFS, recurrence-free survival; ALT, alanine aminotransferase; AFP, α-fetoprotein; HBsAg, hepatitis B surface antigen; CI, confidential interval; HR, hazard ratio. Data obtained from the Cox proportional hazards model; NS, not significant; NA, not adopted. *P* < 0.05 was regarded as statistically significant.

### Prognostic prediction of nomograms for HCC based on KLF4 expression

**Figure 3:**
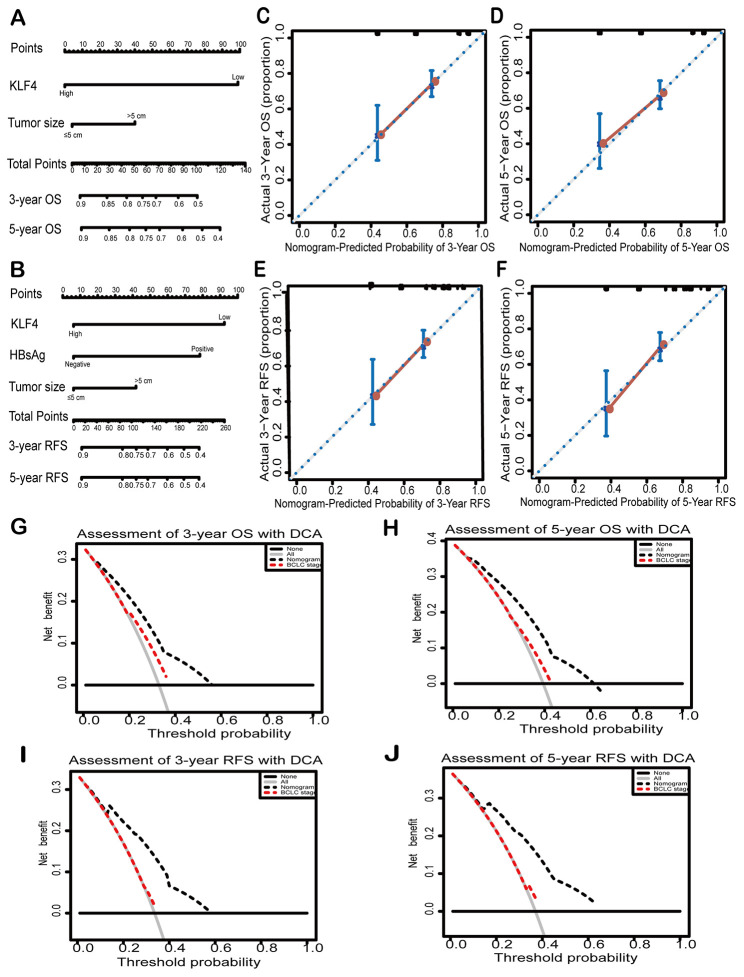
**Nomograms and decision curves analyses were to further study the effect on the prognosis of KLF4.** Pictures (**A**) and (**D**) showed the predictive analysis through prognostic nomogram. First, the plumb line between each factor and the point scale was drawn, and then got the point of each factor. The sum of the points of all factors is the total set of points. finally, a plumb line was drawn from all point scales to the probability scale to obtain the probability of OS or RFS. 3- year and 5- year OS (**B** and **C**) and 3-year and 5- year RFS (**E** and **F**) were shown by calibration curves. The X-axis represented the predicted value of OS or RFS by nomograms and the Y-axis represented actual OS or RFS. The clinical effects of different models were exhibited by decision curve analyses. The comparison of predictions between nomogram-predicted and conventional staging system for 3-year OS and RFS (**G** and **H**) and 5-year OS and RFS was shown (**I** and **J**). BCLC: Barcelona Clinic Liver Cancer staging. Dashed lines: The probability of the clinical net benefit crossing a certain threshold; the solid black horizontal line: to suppose no patients suffer the incident; the gray solid line: to assume all patients suffer the incident.

Two new prognostic nomograms were built based on the results of univariate analysis to predict the OS and RFS of HCC patients, which aimed to further clarify the relationship between KLF4 expression and prognosis of patients (Figure 3A and 3B). As seen in Figure 3C–3F, the calibration curves were used to compare the value of nomograms-prediction and the actual observed value, and found the predicted results is similar to the actual results. The decision curve analyses were drawn to compare the prediction of clinical net benefits between prognostic nomograms and the BCLC staging. According to the comparison, it is found that prognostic nomograms has better prediction capability (Figure 3G–3J). To enhance the predictive accuracy of KLF4 for HCC, C-index (Harrell’s concordance index) was employed to evaluate the performance. Table 3 showed the nomograms for OS and RFS (based on KLF4 expression) owned a better postoperative prediction effect compared to the BCLC staging (***P < 0.001).

**Table 3:** The predicted comparison for OS and RFS between nomogram and BCLC stage in HCC patients.

**Variables**	**Overall survival**		**Recurrence-free survival**	
	**C-index (95% CI)**	***P* value**	**C-index (95% CI)**	***P* value**
BCLC stage	0.563 (0.525-0.601)		0.526 (0.478-0.574)	
Nomogram	0.681 (0.632-0.730)		0.686 (0.634-0.738)	
Nomogram vs. BCLC stage		< 0.001†		< 0.001†

Abbreviations: OS, overall survival; RFS, recurrence-free survival; C-index, concordance index; CI, confidence interval; TNM, Tumor-Nodes-Metastases; BCLC, Barcelona Clinic Liver Cancer.

†: Compared the C-index of nomogram with BCLC stage in patients with HCC.

### KLF4 inhibits HCC cells proliferation

**Figure 4:**
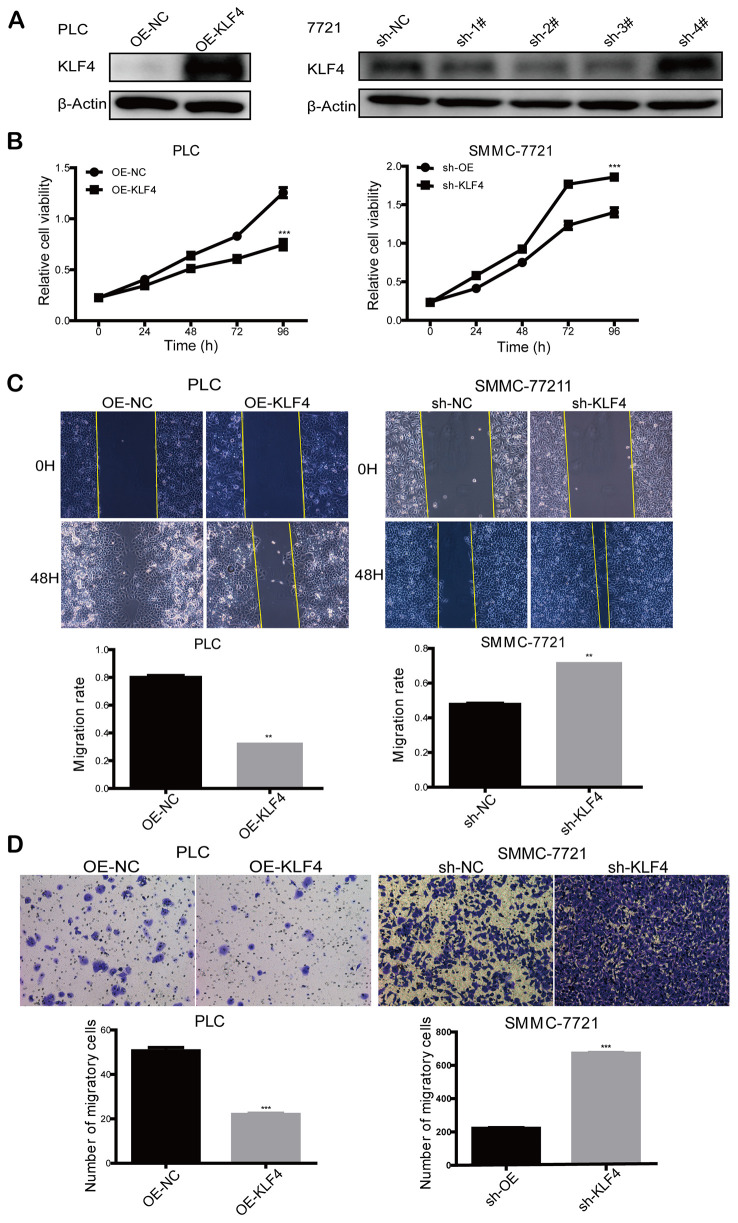
**KLF4 inhibits the proliferation and migration ability of HCC cells.** (**A**) Knockdown of KLF4 in SMMC-7721 and overexpression of KLF4 in PLC/PRF/5 was verified by western blot, and β-actin was used as an internal control in western blot assays. (**B**) CCK8 assay was implemented to detect the proliferation rate of steadily transfected SMMC-7721 and PLC/PRF/5. (**C**) Cell wound scratch assay and (**D**) transwell assay was executed to evaluate the migration rate of steadily transfected SMMC-7721 and PLC/PRF/5. Student’s t-test was used in line charts and bar charts. P < 0.05 was considered statistically significant. * *P* < 0.05, ** *P* < 0.01, *** *P* < 0.001.

Based on the dysregulation of KLF4 expression in HCC patients, we further studied the effects of KLF4 on the biological behaviors of HCC cells. PLC/PRF/5 cells were transfected with pCDHCMV-MCS-EF1-Puro-KLF4 to select stable KLF4 overexpressed cells (OE-KLF4), while SMMC-7721 cells were transfected with pLKO.1-shRNA to select stable KLF4 silenced cells (sh-KLF4). The overexpression control (OE-NC) and shKLF4 control (sh-NC) were transfected with empty plasmids, respectively. The efficiency of overexpression or knockdown of KLF4 in PLC/PRF/5 and SMMC- 7721 cells was detected by Western blot (Figure 4A). We applied the CCK8 assay to detect the cell proliferation rate. As shown in Figure 4B, the KLF4 overexpression could significantly reduce the cell proliferation activity in PLC/PRF/5 cells. While, KLF4 knockdown significantly improved the cell proliferation activity in SMMC- 7721 cells. The results confirmed that KLF4 inhibits cell proliferation of HCC cells in vitro.

### KLF4 inhibits HCC cells migration

Cell wound scratch and transwell assays were employed to explore the effects of KLF4 on cell motility. Compared to the control group, the cell wound scratch assay revealed that over-expressed KLF4 inhibited cell migration in PLC/PRF/5 cells, and knocked-down KLF4 promoted cell migration in SMMC-7721 cells, as shown in Figure 4C. Also, transwell assay showed that over-expressed KLF4 inhibited cell migration in PLC/PRF/5 cells and knocked-down KLF4 promoted cell migration in SMMC-7721 cells in comparison with the respective control group (Figure 4D). Thus, we concluded that KLF4 could inhibit HCC cell migration in vitro.

### KLF4 inhibits the invasion of HCC cells

**Figure 5:**
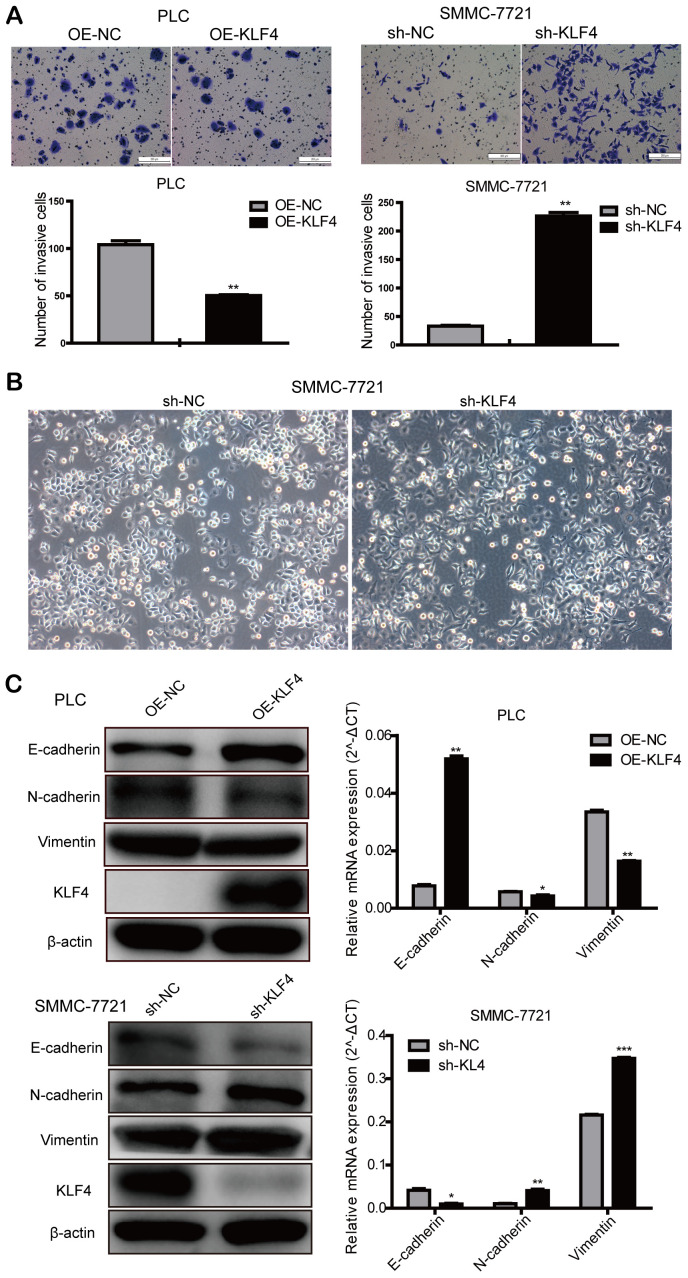
**KLF4 inhibits the invasion of HCC cells.** (**A**) Transwell assay was determined to detect the effect of KLF4 on cell invasion in PLC/PRF/5 and SMMC-7721 cells. Student’s t-test was used in bar charts. (**B**) Changes in cell morphology in SMMC-7721 with shKLF4. (**C**) Western blot and qPCR were used to detect EMT-associated protein expression in steadily transfected PLC/PRF/5 and SMMC-7721 cells. P < 0.05 was considered statistically significant. * *P* < 0.05, ** *P* < 0.01, *** *P* < 0.001.

Transwell assay was used to investigate the effects on invasion by changing KLF4 expression. We found that up-regulated KLF4 expression in PLC/PRF/5 cells inhibited cell invasion, and decreased KLF4 expression in SMMC-7721 cells promoted cell invasion. These results illustrated that KLF4 inhibits the invasion of HCC cells *in vitro* (Figure 5A). Judging from the cellular morphology of SMMC-7721 cells with shKLF4, reduced KLF4 expression leads to the morphological transformation from the epithelial phenotype into the mesenchymal phenotype (Figure 5B). Western blot and qPCR assays showed that KLF4 overexpression in PLC/PRF/5 cells could effectively upregulated the expression of E-cadherin. While KLF4 knockdown could significantly downregulate the expression of N-cadherin and Vimentin (Figure 5C). These results revealed that KLF4 exhibits the capacity to block the activation of EMT pathway.

## DISCUSSION

HCC is one of the most common malignancies and ranks second among the leading causes of cancer-related deaths worldwide, and the morbidity and mortality of men with HCC are two to three times higher than that of women [1]. The low overall survival rate of liver cancer patients may be mainly attributed to cancer recurrence and distant metastasis [2, 27]. Increasing evidence suggested the abnormal expression of KLF4 was detected in many digestive system neoplasms. The point about KLF4 has been supported by a series of research. Shi et al reported that KLF4 repressed lactate dehydrogenase A (LDHA) expression level directly impacting aerobic glycolysis in pancreatic cancer [28]. The research of Zhao WD identified the functional role of KLF4 suppressing colorectal cancer progression [29]. KLF4 could also maintain homeostasis of gastric mucosa, and suppress gastric carcinogenesis and progression [30]. All these findings strengthen the suggestion that KLF4 plays a vital role in suppressing the development and progression in many kinds of tumors. So far, there are ambiguous for the mechanism of HCC progression and no satisfactory prognostic markers for HCC, which highlights the importance of research on the relevant mechanisms in HCC progression and potential biomarkers for HCC patients. Our results suggested KLF4 has the inhibitory effect in HCC progression and is associated with the prognosis of HCC patients receiving curative resection. Our results showed that KLF4 expression is decreased in HCC tissues compared to para-tumorous tissues. Similar results were also observed in the comparison of HCC cell lines with normal liver cell lines. Moreover, the staining results of immunohistochemistry (IHC) showed that KLF4 expression is predominantly decreased in tumor tissues, and lower KLF4 expression was associated with poorer prognosis of HCC patients, which indicated that KLF4 potentially acts as a prognostic marker for HCC patients.

The previous study showed that the transcription factor KLF4 is indispensable in maintaining vascular homeostasis as KLF4 transcriptionally upregulated miR-15a to inhibit angiogenesis vascular endothelial cells [31]. It was reported that sustained KLF4 expression promotes ineffective tumor angiogenesis and diminishes tumor growth [32]. Interestingly, we also observed that KLF4 expression negatively correlated with vascular invasion, suggesting KLF4 may inhibit the tumor growth of HCC by regulating angiogenesis. KLF4 was reported to be a tumor suppressor in colorectal cancer, possibly through elevating the Von Hippel-Lindau gene product, pVHL [33]. In addition, KLF4 transactivated hepatocyte nuclear factor 6 (HNF-6) expression to block the dedifferentiation and progression of hepatocellular carcinoma [34]. Similar results were also observed in our study, which showed that the expression of KLF4 was lower in many types of TCGA tumors, such as LUAD, CHOL and BLCA (when compared to normal tissues). Also, aggressive tumor phenotypes such as advanced BCLC stages, high levels of AFP, present vascular invasion and poor differentiation were associated with low KLF4 expression. These results implied that KLF4 could be considered as a prognostic biomarker in HCC patients.

Research reports that the progression of hepatocellular carcinoma is a stepwise process of dedifferentiation, and well-differentiated HCC commonly appears in the early stage [35]. In our research, we explored the prognostic value of KLF4 in HCC patients through subgroup analysis. According to the analysis, patients with high KLF4 expression had longer OS and RFS in a well-differentiated group of HCC; whereas the correlation between KLF4 expression and prognosis was insignificant in the poorly differentiated subgroup. Patients with poor differentiation may affect the accuracy of KLF4 on prognostic assessment in patients with advanced HCC. Such results indicated KLF4 can act as a potential prognostic biomarker for patients with early HCC. To verify that KLF4 is not only a prognostic biomarker for early HCC patients, we analyzed the OS and RFS of patients in two subgroups of BCLC based on KLF4 expression. The results manifested that patients with high KLF4 expression had prolonged OS and RFS compared with the patients with low KLF4 expression in two subgroups of BCLC. According to univariate and multivariate analysis, we concluded KLF4 was an independent prognostic factor regardless of HCC stage, tumor differentiation, and tumor size. Furthermore, the prognostic nomogram model based on KLF4 expression was employed to prove the prediction accuracy of KLF4 for HCC patients’ OS and RFS. Then, we further studied the effects of KLF4 on the biological behaviors of HCC cells. Western blot and qPCR were used to detect KLF4 expression in HCC cell lines. Results show KLF4 expression in most HCC cells (PLC/PRF/5, Hep3B, Huh7, HepG2) was lower, while in SMMC- 7721 cells was higher when compared to the normal liver cells (L02). Considering KLF4 expression levels in HCC cell lines, PLC/PRF/5 cells were selected to overexpress exogenous KLF4, and SMMC-7721 cells were transfected with shRNA of KLF4 to explore the effects of KLF4 on the biological behaviors of HCC cells. A series of in vitro study revealed that KLF4 overexpression inhibited cell proliferation, migration, and invasion of PLC/PRF/5 cells while KLF4 knockdown enhanced such activities in SMMC-7721 cells, which supported KLF4’s antitumor function in HCC. Mounting research has shown that EMT plays a significant role in acquiring the capability of migration and invasion for cancer cell in cancer progression and metastasis[36, 37]. In addition, judging from the cellular morphology of SMMC-7721 cells with shKLF4, reduced KLF4 expression leads to the morphological transformation from the epithelial phenotype into the mesenchymal phenotype. Moreover, elevated KLF4 expression upregulated E-cadherin expression and decreased N-cadherin, Vimentin expression in PLC/PRF/5 cells, While reduced KLF4 expression downregulated E-cadherin expression and elevated N-cadherin, Vimentin expression in SMMC-7721 cells. These results demonstrated that KLF4 exhibits the capacity to inhibit HCC progression by blocking the activation of EMT pathway. Thereby, we regarded KLF4 as a tumor suppressor that could inhibit HCC progression.

However, there were some limitations in our research. Firstly, our results need to be further verified in a larger cohort of the patients considering the limited number of patients enrolled in this study. Secondly, this study was a retrospective analysis. Therefore, future prospective analysis to validate the results is needed. An independent cohort will also be needed to validate the findings, and the molecular mechanism of KLF4 inhibiting EMT in HCC progression should be further explored in future study.

In summary, we illustrated clearly that KLF4 as a suppressor can block the activation of EMT in HCC progression. And KLF4 is downregulated in HCC tumors and can be regarded as a prognostic factor to predict OS and RFS of HCC patients after curative resection. In addition, the nomograms in this research can further improve the prediction for OS and RFS of HCC patients by integrating KLF4 and other independent clinical factors. Hence, KLF4 may be a potential therapeutic biomarker and prognostic indicator for HCC patients. The antitumor effect of KLF4 and the functional mechanisms need to be explored in future studies.

## MATERIALS AND METHODS

### Patient selected and follow-up

We completed a statistical analysis of registered information from 185 HCC patients in Zhongshan Hospital, Fudan University. All patients had a hepatectomy from July 2010 to July 2011. Patients' screening criteria were as follows: patients did not receive any systemic or local treatments nor experience extrahepatic metastases before the operation. All patients who underwent primary and therapeutic hepatectomy had a definite postoperative pathological diagnosis of HCC. These patients who all showed absolute follow-up data and clinicopathological features had no infection or inflammatory response other than viral hepatitis. The patients underwent a series of examinations such as alanine aminotransferase (ALT), liver cirrhosis, hepatitis B virus surface antigens (HBsAg), alpha-fetoprotein (AFP), tumor encapsulation, tumor number, tumor size, vascular invasion, differentiated degree, and Barcelona Clinic Liver Cancer staging (BCLC) within 3 days before the operation. All patients were followed up and received blood routine examination, blood biochemical examination, serological examination of tumor markers, abdominal ultrasound, and chest imaging examination. The follow-up time was defined as every three months for the first five years after surgery and once a year for the time after. The deadline for data collection was July 2018 or the time of death in deceased patients. The study was conducted with the patients’ knowledge and permitted by the hospital ethics committee. The academic research was conducted under ethical standards.

### Tissue samples and immunohistochemistry

The human HCC tissues microarray was made of the tumor tissues and para-carcinoma tissues collected from the 185 HCC patients after curative resection. We then sorted out and analyzed the corresponding information of tissue microarray. Tissue microarrays were constructed with surgical specimens fixed by formalin and embedded by paraffin. Anti-KLF4 antibody (ab215036; Abcam) was used for incubating sections. Immunohistochemical analysis was performed according to the previous method [38]. Then, immunohistochemical results were assessed by two independent pathologists who did not have access to patients’ information. The staining degree of KLF4 was scored semi-quantitatively as 3 for strong, 2 for moderate, 1 for weak, 0 for negative. The score of the staining area was calculated by the percentage of stained positive cells and was scored as 4 for >75%, 3 for 51%- 75%, 2 for 26%- 50%,1 for 5%- 25%, 0 for <5%. The final results described each specimen through multiplying the staining degree score by the staining area score. The expression level of KLF4 in each specimen was evaluated according to the score value with 9- 12 as a strong level, 6- 8 as a moderate level, 1- 4 as a weak level, 0 as a negative level.

### Cell culture and transfection

There are six types of cells (five types of liver cancer cells: SMMC- 7721, HepG2, Hep3B, Huh7, and PLC/PRF/5, and a normal liver cell (L02)) were included in the experiments and all cell identification reports were provided in supplementary materials. All these cells were acquired from the cell bank of the Chinese Academy of Sciences (Shanghai, China). All the cells were cultured with Dulbecco's Modified Eagle Medium (DMEM basic; Gibco). The concentration of fetal bovine serum (FBS; Gibco) in DMEM was 10%. These cells were then cultured at cell incubator (Thermo; America) with 5% CO2 at 37°C. Lentiviral particles loaded the KLF4 overexpression plasmid (OE-KLF4) to infect PLC/PRF/5 cells, empty vectors (OE-NC) were used as overexpression control. SMMC- 7721 cells were infected with lentiviral particles, loading shRNA-KLF4 plasmid (sh-KLF4), and empty vector (sh-NC) as shRNA control.

### RNA extraction and quantitative reverse transcription PCR (qRT-PCR)

Firstly, PBS was used to wash the collected HCC cells. Then, total RNA was extracted with the TRIzol reagent (Invitrogen, USA). Next, 1 μg of total RNA was used for reverse transcription with Reverse Transcription Reagent Kit (Takara, Japan). The mRNA expression of KLF4 was detected with TB Green qPCR Mix (Takara, Japan) on the basis of reagent supplying protocols. The primers were used for detection as follows: KLF4-forward: 5′-CAAGTCCCGCCGCTCCATTA-3′; KLF4-reverse: 5′-CCATCCACAGCCGTCCCAGT-3′; β-actin-forward: 5′-GGACCTGACTGACTACCTCAT-3′; β-actin-reverse: 5′-CGTAGCACAGCTTCTCCTTAAT-3′; E-cadherin-forward: 5′-CGAGAGCTACACGTTCACGG-3′; E-cadherin-reverse: 5′-GGGTGTCGAGGGAAAAATAGG-3′; N-cadherin-forward: 5′-TGCGGTACAGTGTAACTGGG-3′; N-cadherin-reverse: 5′-GAAACCGGGCTATCTGCTCG-3′; Vimentin-forward: 5′-CGGGAGAAATTGCAGGAGGA-3′; Vimentin-reverse: 5′-AAGGTCAAGACGTGCCAGAG-3′. The conditions of the experimental reaction were: 1 cycle for 30 seconds (95 °C),40 cycles of denaturation for 5 seconds (95 °C), and annealing for 34 seconds (60°C). Finally, the gene expression differences were analyzed according to the experimental results. Three repetitions were performed in the experiment.

### Western blot

The protein expression level of KLF4 in the HCC cell lines was detected by Western blot. RIPA lysis buffer was employed to extract total protein of HCC cells, and then 10% SDS- PAGE were used to separate protein samples. The PVDF membrane (IPVH 00010; Millipore) with a pore size of 0.45μm was used to transfer the cell protein. After 5% defatted milk blocking for 90 minutes at room temperature, anti-KLF4 antibody (ab215036; Abcam) and anti-β-actin antibody (#13E5; CST) were used for incubating the membranes at 4°C overnight. On the second day, TBST (1% Tween diluted in TBS) was used to wash the membranes, then the membranes were incubated with diluted secondary antibodies (AP132P; EMD Millipore) at room temperature for 90 minutes. Finally, the membranes were scanned with equipment (Image Quant LAS 4000; Sweden).

### Cell proliferation assay

We employed CCK8 assay (http:www.dojingdo.cn) to detect the cell proliferation activity and viability of the selected SMMC- 7721 and PLC/PRF/5 cells. Counted cells were cultured with 96-well plates at 37 °C. The absorbance at 450nm was observed at different time points with each well injected into the 10ul CCK8 kit so that we can compare the rate of cell proliferation by the value of absorbance. Each well has three identical wells.

### Cell migration and invasion assays

For cell wound scratch experiment, selected cells and cells of the control group were cultured in 6-well plates. Each well was covered with cells and scratched with white tips on the next day. Then pictures were taken at different time points. Cell migration and invasion was examined by transwell assays. Matrigel matrix basement membrane (#354234; Corning) was added into the chambers (8.0μm pores; Corning) before culturing cells in transwell invasion assay. For transwell migration and invasion assays, a certain amount of medium with serum was added to 24-well plate, then chambers were placed on the plate. The counted cells were cultured with serum-free DMEM in chambers for a few hours, where they can migrate through the holes planting on the membranes of the chambers. Finally, the cells on the membranes were fixed with 4% paraformaldehyde and the fixed cells stained with crystal violet. The average number of cells in the five fields was regarded as the number of migrating cells.

### Statistical analysis

Using the TCGA database, we estimated the differential expression of KLF4 between tumor tissues and para-tumorous tissues in different types of cancers with the method of the Wilcoxon test. The results were shown in box plots. The relationship between clinicopathologic features and KLF4 was evaluated by student’s t-test and Pearson chi-square test. The OS and RFS were assessed by the Log-rank test and Kaplan Meier survival analysis. For other data, they were evaluated by means ± standard deviation (SD), and SPSS 19.0 was used to complete the analysis. According to the distribution of data, student’s t-test or Mann-Whitney U test was used for the comparison of differences between groups. The Cox proportional risk regression model was employed to perform univariate and multivariate analyses. The property of a nomogram was estimated according to three ways: the decision curve analysis (DCA), calibration curve and Concordance index (C- index). Only when *P < 0.05, results reached statistical significance.

## References

[1] Bray F, Ferlay J, Soerjomataram I, Siegel RL, Torre LA, Jemal A. Global cancer statistics 2018: GLOBOCAN estimates of incidence and mortality worldwide for 36 cancers in 185 countries. CA Cancer J Clin. 2018; 68:394–424. 10.3322/caac.21492 30207593

[2] Chen W, Zheng R, Baade PD, Zhang S, Zeng H, Bray F, Jemal A, Yu XQ, He J. Cancer statistics in China, 2015. CA Cancer J Clin. 2016; 66:115–32. 10.3322/caac.21338 26808342

[3] Yang JD, Roberts LR. Hepatocellular carcinoma: a global view. Nat Rev Gastroenterol Hepatol. 2010; 7:448–58. 10.1038/nrgastro.2010.100 20628345 PMC3926946

[4] Llovet JM, Burroughs A, Bruix J. Hepatocellular carcinoma. Lancet. 2003; 362:1907–17. 10.1016/S0140-6736(03)14964-1 14667750

[5] Kulik L, El-Serag HB. Epidemiology and management of hepatocellular carcinoma. Gastroenterology. 2019; 156:477–91.e1. 10.1053/j.gastro.2018.08.065 30367835 PMC6340716

[6] European Association for the study of the Liver, and European Organisation for Research and Treatment of Cancer. EASL-EORTC clinical practice guidelines: management of hepatocellular carcinoma. J Hepatol. 2012; 56:908–43. 10.1016/j.jhep.2011.12.001 22424438

[7] Bruix J, Takayama T, Mazzaferro V, Chau GY, Yang J, Kudo M, Cai J, Poon RT, Han KH, Tak WY, Lee HC, Song T, Roayaie S, et al. Adjuvant sorafenib for hepatocellular carcinoma after resection or ablation (STORM): a phase 3, randomised, double-blind, placebo-controlled trial. Lancet Oncol. 2015; 16:1344–54. 10.1016/S1470-2045(15)00198-9 26361969

[8] Palmer DH. Sorafenib in advanced hepatocellular carcinoma. N Engl J Med. 2008; 359:2498. 19065750

[9] Cheng AL, Kang YK, Chen Z, Tsao CJ, Qin S, Kim JS, Luo R, Feng J, Ye S, Yang TS, Xu J, Sun Y, Liang H, et al. Efficacy and safety of sorafenib in patients in the Asia-pacific region with advanced hepatocellular carcinoma: a phase III randomised, double-blind, placebo-controlled trial. Lancet Oncol. 2009; 10:25–34. 10.1016/S1470-2045(08)70285-7 19095497

[10] Kudo M, Han G, Finn RS, Poon RT, Blanc JF, Yan L, Yang J, Lu L, Tak WY, Yu X, Lee JH, Lin SM, Wu C, et al. Brivanib as adjuvant therapy to transarterial chemoembolization in patients with hepatocellular carcinoma: a randomized phase III trial. Hepatology. 2014; 60:1697–707. 10.1002/hep.27290 24996197

[11] Ghaleb AM, Yang VW. Krüppel-like factor 4 (KLF4): what we currently know. Gene. 2017; 611:27–37. 10.1016/j.gene.2017.02.025 28237823 PMC5391259

[12] Farrugia MK, Vanderbilt DB, Salkeni MA, Ruppert JM. Kruppel-like pluripotency factors as modulators of cancer cell therapeutic responses. Cancer Res. 2016; 76:1677–82. 10.1158/0008-5472.CAN-15-1806 26964625 PMC4873413

[13] Tetreault MP, Yang Y, Katz JP. Krüppel-like factors in cancer. Nat Rev Cancer. 2013; 13:701–13. 10.1038/nrc3582 24060862

[14] Evans PM, Liu C. Roles of krüpel-like factor 4 in normal homeostasis, cancer and stem cells. Acta Biochim Biophys Sin (Shanghai). 2008; 40:554–64. 10.1111/j.1745-7270.2008.00439.x 18604447 PMC2668950

[15] Yang WT, Zheng PS. Krüppel-like factor 4 functions as a tumor suppressor in cervical carcinoma. Cancer. 2012; 118:3691–702. 10.1002/cncr.26698 22170594

[16] Jia Y, Ying X, Zhou J, Chen Y, Luo X, Xie S, Wang QC, Hu W, Wang L. The novel KLF4/PLAC8 signaling pathway regulates lung cancer growth. Cell Death Dis. 2018; 9:603. 10.1038/s41419-018-0580-3 29789534 PMC5964121

[17] Zhang J, Zhu Z, Wu H, Yu Z, Rong Z, Luo Z, Xu Y, Huang K, Qiu Z, Huang C. PODXL, negatively regulated by KLF4, promotes the EMT and metastasis and serves as a novel prognostic indicator of gastric cancer. Gastric Cancer. 2019; 22:48–59. 10.1007/s10120-018-0833-y 29748877 PMC6314994

[18] Ghaleb AM, Nandan MO, Chanchevalap S, Dalton WB, Hisamuddin IM, Yang VW. Krüppel-like factors 4 and 5: the yin and yang regulators of cellular proliferation. Cell Res. 2005; 15:92–96. 10.1038/sj.cr.7290271 15740636 PMC1317089

[19] Yan Y, Li Z, Kong X, Jia Z, Zuo X, Gagea M, Huang S, Wei D, Xie K. KLF4-mediated suppression of CD44 signaling negatively impacts pancreatic cancer stemness and metastasis. Cancer Res. 2016; 76:2419–31. 10.1158/0008-5472.CAN-15-1691 26880805 PMC4876033

[20] Yu F, Li J, Chen H, Fu J, Ray S, Huang S, Zheng H, Ai W. Kruppel-like factor 4 (KLF4) is required for maintenance of breast cancer stem cells and for cell migration and invasion. Oncogene. 2011; 30:2161–72. 10.1038/onc.2010.591 21242971 PMC3088782

[21] Yang X, Zhang D, Liu S, Li X, Hu W, Han C. KLF4 suppresses the migration of hepatocellular carcinoma by transcriptionally upregulating monoglyceride lipase. Am J Cancer Res. 2018; 8:1019–29. 30034939 PMC6048399

[22] Li Q, Song W, Wang W, Yao S, Tian C, Cai X, Wang L. Suppression of epithelial-mesenchymal transition in hepatocellular carcinoma cells by krüppel-like factor 4. Oncotarget. 2016; 7:29749–60. 10.18632/oncotarget.8831 27102441 PMC5045430

[23] Savagner P, Boyer B, Valles AM, Jouanneau J, Thiery JP. Modulations of the epithelial phenotype during embryogenesis and cancer progression. Cancer Treat Res. 1994; 71:229–49. 10.1007/978-1-4615-2592-9_12 7946950

[24] Nieto MA, Huang RY, Jackson RA, Thiery JP. Emt: 2016. Cell. 2016; 166:21–45. 10.1016/j.cell.2016.06.028 27368099

[25] Cui J, Shi M, Quan M, Xie K. Regulation of EMT by KLF4 in gastrointestinal cancer. Curr Cancer Drug Targets. 2013; 13:986–95. 10.2174/15680096113136660104 24168184 PMC4127075

[26] Yori JL, Johnson E, Zhou G, Jain MK, Keri RA. Kruppel-like factor 4 inhibits epithelial-to-mesenchymal transition through regulation of e-cadherin gene expression. J Biol Chem. 2010; 285:16854–63. 10.1074/jbc.M110.114546 20356845 PMC2878056

[27] Tang A, Hallouch O, Chernyak V, Kamaya A, Sirlin CB. Epidemiology of hepatocellular carcinoma: target population for surveillance and diagnosis. Abdom Radiol (NY). 2018; 43:13–25. 10.1007/s00261-017-1209-1 28647765

[28] Shi M, Cui J, Du J, Wei D, Jia Z, Zhang J, Zhu Z, Gao Y, Xie K. A novel KLF4/LDHA signaling pathway regulates aerobic glycolysis in and progression of pancreatic cancer. Clin Cancer Res. 2014; 20:4370–80. 10.1158/1078-0432.CCR-14-0186 24947925 PMC4134726

[29] Zhao W, Hisamuddin IM, Nandan MO, Babbin BA, Lamb NE, Yang VW. Identification of krüppel-like factor 4 as a potential tumor suppressor gene in colorectal cancer. Oncogene. 2004; 23:395–402. 10.1038/sj.onc.1207067 14724568 PMC1351029

[30] Wei D, Gong W, Kanai M, Schlunk C, Wang L, Yao JC, Wu TT, Huang S, Xie K. Drastic down-regulation of krüppel-like factor 4 expression is critical in human gastric cancer development and progression. Cancer Res. 2005; 65:2746–54. 10.1158/0008-5472.CAN-04-3619 15805274

[31] Zheng X, Li A, Zhao L, Zhou T, Shen Q, Cui Q, Qin X. Key role of microRNA-15a in the KLF4 suppressions of proliferation and angiogenesis in endothelial and vascular smooth muscle cells. Biochem Biophys Res Commun. 2013; 437:625–31. 10.1016/j.bbrc.2013.07.017 23867820

[32] Hale AT, Tian H, Anih E, Recio FO 3rd, Shatat MA, Johnson T, Liao X, Ramirez-Bergeron DL, Proweller A, Ishikawa M, Hamik A. Endothelial kruppel-like factor 4 regulates angiogenesis and the notch signaling pathway. J Biol Chem. 2014; 289:12016–28. 10.1074/jbc.M113.530956 24599951 PMC4002108

[33] Gamper AM, Qiao X, Kim J, Zhang L, DeSimone MC, Rathmell WK, Wan Y. Regulation of KLF4 turnover reveals an unexpected tissue-specific role of pVHL in tumorigenesis. Mol Cell. 2012; 45:233–43. 10.1016/j.molcel.2011.11.031 22284679 PMC3982234

[34] Sun H, Tang H, Xie D, Jia Z, Ma Z, Wei D, Mishra L, Gao Y, Zheng S, Xie K, Peng Z. Krüppel-like factor 4 blocks hepatocellular carcinoma dedifferentiation and progression through activation of hepatocyte nuclear factor-6. Clin Cancer Res. 2016; 22:502–12. 10.1158/1078-0432.CCR-15-0528 26338995 PMC4715982

[35] Kojiro M. Histopathology of liver cancers. Best Pract Res Clin Gastroenterol. 2005; 19:39–62. 10.1016/j.bpg.2004.10.007 15757804

[36] Aiello NM, Kang Y. Context-dependent EMT programs in cancer metastasis. J Exp Med. 2019; 216:1016–26. 10.1084/jem.20181827 30975895 PMC6504222

[37] Krebs AM, Mitschke J, Lasierra Losada M, Schmalhofer O, Boerries M, Busch H, Boettcher M, Mougiakakos D, Reichardt W, Bronsert P, Brunton VG, Pilarsky C, Winkler TH, et al. The EMT-activator Zeb1 is a key factor for cell plasticity and promotes metastasis in pancreatic cancer. Nat Cell Biol. 2017; 19:518–29. 10.1038/ncb3513 28414315

[38] Dong Q, Zhu X, Dai C, Zhang X, Gao X, Wei J, Sheng Y, Zheng Y, Yu J, Xie L, Qin Y, Qiao P, Zhou C, et al. Osteopontin promotes epithelial-mesenchymal transition of hepatocellular carcinoma through regulating vimentin. Oncotarget. 2016; 7:12997–3012. 10.18632/oncotarget.7016 26824421 PMC4914337

