# Correction for: The association between KLF4 as a tumor suppressor and the prognosis of hepatocellular carcinoma after curative resection

**DOI:** 10.18632/aging.206197

**Published:** 2025-01-31

**Authors:** Min Xue, Chenhao Zhou, Yan Zheng, Ziping Zhang, Shun Wang, Yan Fu, Manar Atyah, Xiaolong Xue, Le Zhu, Qiongzhu Dong, Huliang Jia, Ning Ren, Ruolei Hu

**Affiliations:** 1 Department of Biochemistry and Molecular Biology, Laboratory of Molecular Biology, Anhui Medical University, Hefei, China; 2 Department of Liver Surgery, Liver Cancer Institute, Zhongshan Hospital, Fudan University, Shanghai, China; 3 Department of General Surgery, Huashan Hospital and Cancer Metastasis Institute, Fudan University, Shanghai, China; 4 Institute of Fudan Minhang Academic Health System, Minhang Hospital, Fudan University, Shanghai, China

**Keywords:** KLF4, hepatocellular carcinoma, prognosis, overall survival, recurrence-free survival

**This article has been corrected:** The authors recently found that there was overlap between two IHC images in **Figure 1E**. Specifically, the image representing weak KLF4 staining of HCC tumor tissues was unintentionally misused for the image from the negative staining group. The authors replaced incorrect image with the original image from the KLF4 weak staining group and stated that this correction has no impact on the experimental outcome or conclusions. The authors sincerely apologize for any inconvenience caused.

The corrected version of **Figure 1** is provided below.

**Figure 1:**
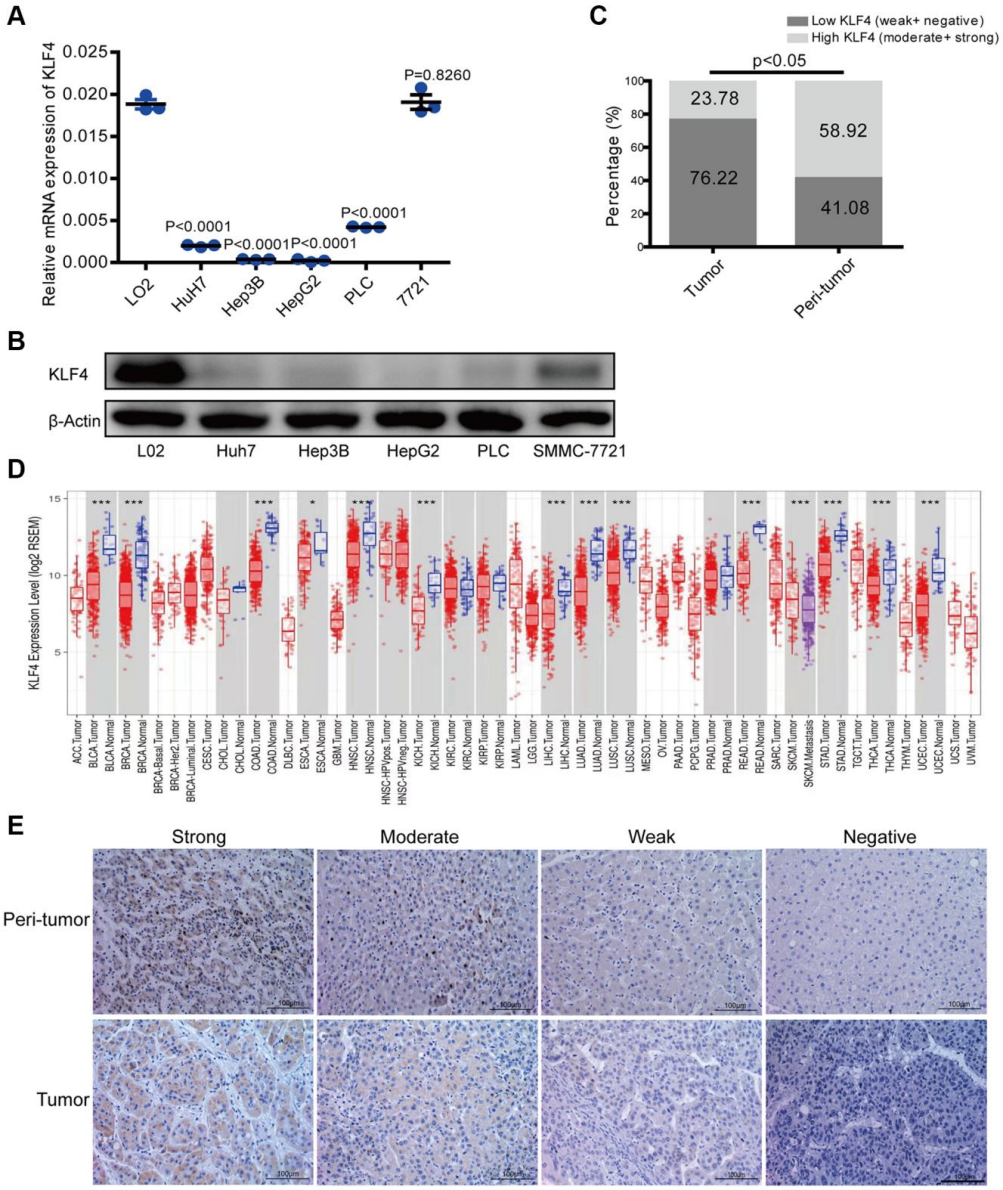
**KLF4 expression in hepatocellular carcinoma (HCC) tissues and cell lines.** (**A**) KLF4 expression was detected in the mRNA level among five HCC cell lines and one normal liver cell (L02). (**B**) KLF4 expression was detected in the protein level in six cell lines. The internal control was β-actin. (**C**) Immunohistochemical results were analyzed by chi-square test to compare the distribution of KLF4 in HCC tumors and adjacent tissues. (**D**) KLF4 expression was analyzed in tumor and para-tumorous tissues in TCGA tumors. (**E**) KLF4 expression was exhibited through characteristic photos of immunostaining in HCC tumor and para-tumorous tissues. Image scale = 100 μm. *P* < 0.05 was considered statistically significant, ^*^P < 0.05, ^**^P < 0.01, ^***^P < 0.001.

